# Diacylglycerol Kinase β Knockout Mice Exhibit Attention-Deficit Behavior and an Abnormal Response on Methylphenidate-Induced Hyperactivity

**DOI:** 10.1371/journal.pone.0037058

**Published:** 2012-05-10

**Authors:** Mitsue Ishisaka, Kenichi Kakefuda, Atsushi Oyagi, Yoko Ono, Kazuhiro Tsuruma, Masamitsu Shimazawa, Kiyoyuki Kitaichi, Hideaki Hara

**Affiliations:** 1 Molecular Pharmacology, Department of Biofunctional Evaluation, Gifu Pharmaceutical University, Gifu, Japan; 2 Department of Pharmacy, Gifu University Hospital, Gifu, Japan; Tokyo Metropolitan Institute of Medical Science, Japan

## Abstract

**Background:**

Diacylglycerol kinase (DGK) is an enzyme that phosphorylates diacylglycerol to produce phosphatidic acid. DGKβ is one of the subtypes of the DGK family and regulates many intracellular signaling pathways in the central nervous system. Previously, we demonstrated that DGKβ knockout (KO) mice showed various dysfunctions of higher brain function, such as cognitive impairment (with lower spine density), hyperactivity, reduced anxiety, and careless behavior. In the present study, we conducted further tests on DGKβ KO mice in order to investigate the function of DGKβ in the central nervous system, especially in the pathophysiology of attention deficit hyperactivity disorder (ADHD).

**Methodology/Principal Findings:**

DGKβ KO mice showed attention-deficit behavior in the object-based attention test and it was ameliorated by methylphenidate (MPH, 30 mg/kg, i.p.). In the open field test, DGKβ KO mice displayed a decreased response to the locomotor stimulating effects of MPH (30 mg/kg, i.p.), but showed a similar response to an *N*-methyl-d-aspartate (NMDA) receptor antagonist, MK-801 (0.3 mg/kg, i.p.), when compared to WT mice. Examination of the phosphorylation of extracellular signal-regulated kinase (ERK), which is involved in regulation of locomotor activity, indicated that ERK1/2 activation induced by MPH treatment was defective in the striatum of DGKβ KO mice.

**Conclusions/Significance:**

These findings suggest that DGKβ KO mice showed attention-deficit and hyperactive phenotype, similar to ADHD. Furthermore, the hyporesponsiveness of DGKβ KO mice to MPH was due to dysregulation of ERK phosphorylation, and that DGKβ has a pivotal involvement in ERK regulation in the striatum.

## Introduction

Stimulation of cell surface Gq protein-coupled receptors by many extracellular stimuli such as growth factors, hormones, and neurotransmitters, activates phospholipase C and results in the production of diacylglycerol (DG) from inositol phospholipids [1]. DG then directly binds to protein kinase C, activating this multifunctional enzyme [2]. DG also activates other proteins such as Ras guanyl nucleotide-releasing proteins [3]. DG is further converted to phosphatidic acid (PA), and PA in turn also activates many other proteins, such as mammalian target of rapamycin and Raf-1 kinase [4]. The conversion of DG to PA is catalyzed by diacylglycerol kinase (DGK) [5]; that is, DGK controls the functional balance of these two lipid mediators by catabolizing DG and producing PA.

To date, ten DGK isozymes have been identified from mammalian cells [6], [7], [8]. Among these, DGKβ is classified as a type I DGK, as it possesses a calcium-binding EF-hand [9]. DGKβ is widely distributed in the brain, particularly in the olfactory bulb, cerebral cortex, striatum, and hippocampus, all of which are areas that correspond to the dopaminergic projection field [10]. In addition, coexpression of DGKβ with dopamine D1 and D2 receptors has been reported in medium spiny neurons of the striatum, suggesting a connection between DGKβ and dopaminergic transmission [11].

Dysfunction in dopamine signaling has been implicated in many neuropsychiatric disorders, such as Parkinson's disease, schizophrenia, attention deficit hyperactivity disorder (ADHD), and drug abuse. Analysis of the human DGKβ gene has demonstrated that a splice variant at the COOH-terminal of DGKβ is related to bipolar disorder [12]. Related to this, we have reported that DGKβ knockout (KO) mice show mania-like behavior which is attenuated by lithium treatment [13]. We and other researchers have also reported that spine formation was regulated by the control of lipid levels by DGKβ and that DGKβ KO mice showed impaired memory [14], [15]. These findings suggest that DGKβ is directly involved in the regulation of psychomotor behavior and higher brain functions.

ADHD is a heterogeneous developmental syndrome characterized by inattention, impulsivity, and hyperactivity. It had been reported that a majority of children, adolescents, and adults with ADHD are affected by other comorbid psychiatric conditions, such as bipolar disorders [16], [17]. We made a hypothesis that DGKβ which is suspected of contributing to bipolar disorder, also plays some important roles in ADHD.

Methylphenidate (MPH) is a psychostimulant drug approved for treatment of ADHD, postural orthostatic tachycardia syndrome, and narcolepsy. Some reports have indicated that MPH treatment significantly decreases the hyperactive locomotor activity of mice with dopamine transporter KO [18], casein kinase Iδ overexpression [19], or p35 KO [20].

In the present study, we further analyzed the behavior of DGKβ KO mice using some behavioral tests and a psychostimulant MPH in order to investigate the function of DGKβ in the central nervous system, especially in the pathophysiology of ADHD.

## Materials and Methods

### Animals

DGKβ KO mice (C57BL/6N) were generated using the *Sleeping Beauty* transposon system as described in our previous report [14]. Wild-type (WT) and DGKβ KO mice were generated by breeding heterozyrous mutants, and we used WT littermates as a control group of DGKβ KO mice. The animals (8–20 weeks old) were housed at 24±2°C under a 12 hrs light-dark cycle (lights on from 8:00 to 20:00) and had ad libitum to food and water. These studies were approved by the Animal Experiment Committee of Gifu Pharmaceutical University (permission number; 2009-061, 2009-331, 2009-378, 2010-396, 2010-398, 2011-067, 2011-083, 2011-283, and 2011-416). All procedures relating to animal care and treatment conformed to the animal care guidelines of this committee. All efforts were made to minimize both suffering and the number of animals used. Only aged-matched male mice were used for behavioral experiments, and all other tests included age-matched males and females in proportional contribution across groups. Behavioral experiments were performed during the light phase, between 10:00 and 18:00. To avoid stress-interference in the behavioral tests, mice were not repeatedly used.

### Object-based attention test

The experiment were performed based on a previous report [21]. The apparatus is a rectangular, two-chambered boxes including exploring chamber (length 30×width 30×height 22 cm) and test chamber (length 30×width 15×height 22 cm). The chambers were always bedded with fresh animal bedding for every mouse after the floors of chambers were wiped with tissue paper (70% alcohol). The experiments were divided into three different phases: a habituation phase, an acquisition phase, and a retention phase. On the habituation phase, mice were individually subjected to a single familiarization session of 10 min, during which they were exposed to both empty chambers. On the acquisition phase, animals were subjected to a single 3-min or 6-min session, during which five objects (A, B, C, D, and E) were placed separately in the exploring chamber. All objects were made of the same wooden material with the similar color and smell, but different in shape. On the retention phase, one of the object in the exploring chamber (object B) was placed into the test chamber in parallel with the novel F object, which was also made of the same wooden material with the similar color and smell, but different in shape with other objects. Then the mouse was immediately allowed to enter the test chamber (within 10 s) and to explore two objects (object B and F) for 3 min. For the analysis, all sessions were recorded using video camera, and the time spent exploring each object was measured using stopwatch. A recognition index of retention session was expressed as the ratio (TF×100)/(TB+TF), where TB and TF are the time spent on object B and object F, respectively.

To evaluate the effect of MPH on the attention, vehicle (saline) or MPH (30 mg/kg) was intraperitoneally injected (i.p.) 20 min before habituation phase.

### Open field test

For the assessment of the rearing behavior in the novel environment, each mouse was placed in the periphery of the open field apparatus (length 30×width 30×height 30 cm), and mouse behavior was monitored for 5 min. The number of rearing actions was counted manually.

For measurement of the effects of psychostimulants on locomotor activity, each mouse was placed in the open field apparatus. Thirty minutes later, MPH (0.3, 3, or 30 mg/kg), MK-801 (0.3 mg/kg), or vehicle (saline) was injected (i.p.), the mouse was returned to the apparatus, and monitored for a further 90 min after injection. The total distance moved in the arena was recorded using a computer-operated EthoVision XT system (Noldus, Wageningen, the Netherlands). Stereotypies and rearing were analyzed manually by an observer blind to the treatment for 5 min. Stereotype behaviors were defined as sniffing while immobile, grooming, licking the bottom of wall of the box. The total duration of stereotyped behaviors and the number of rearing were analyzed.

### Catalepsy test

Time spent in a cataleptic position was monitored by positioning the mouse so that both front paws rested on a 0.3 cm diameter pole 3.5 cm above a bench surface. The time for which each mouse maintained this position was recorded up to a maximum of 2 min. Haloperidol (Sigma Aldrich, St. Louis, MO, USA) dissolved in 0.3% tartaric acid was administered i.p. and catalepsy was measured 1 h after haloperidol administration, and three times in each test session.

### Immunostaining

Mice were anesthetized with sodium pentobarbital (50 mg/kg, i.p.) (Nembutal; Dainippon-Sumitomo Pharmaceutical Co. Ltd., Osaka, Japan) and brains were perfusion-fixed with 4% paraformaldehyde (Wako Pure Chemical Industries, Osaka, Japan) in 0.1 M phosphate buffer (pH 7.4). The brains were removed after a 20-min perfusion fixation at 4°C, then immersed in the same fixative solution overnight at 4°C. The brains were then immersed in 25% sucrose (Wako) in 0.1 M phosphate buffer for 24 h, and frozen. Coronal brain sections (14 µm) were cut on a cryostat and placed on slides (MASCOAT; Matsunami, Osaka, Japan). The sections were washed for 5 min in 0.01 M phosphate buffered saline (PBS), then treated with 0.3% hydrogen peroxidase (Wako) in 10% methanol (Wako). They were then washed three times in 0.01 M PBS, followed by a 30-min pre-incubation with 10% normal goat serum. The sections were then incubated with anti-tyrosine hydroxylase (TH) antibody (1∶200 dilution; Millipore, Billerica, MA, USA), including 0.3% triton X-100 (Nacalai tesque, Kyoto, Japan), for 3 h at 4°C. After a 15-min rinse in changes of 0.01 M PBS, the sections were incubated with biotinylated second rabbit antibody for 2 h, and then with an avidin-biotin peroxidase complex (ABC Elite kit; Vector Laboratories, Peterborough, UK) for 30 min (both at room temperature). Optical density measurements of striatal TH immunostaining were obtained by digital image analysis (Scion, Frederick, MD, USA).

### Western blot analysis

Each mouse was decapitated and the brain was quickly removed from the skull, briefly washed in ice-cold saline, and laid on a cooled (4°C) metal plate, on which the brain was rapidly dissected to separate the striatum, hippocampus, and prefrontal cortex. To extract protein, the tissue was homogenized in cell-lysis buffer using a homogenizer (Physcotron; Microtec Co. Ltd., Chiba, Japan). The lysate was centrifuged at 12,000× g for 20 min and the supernatant used for this study. The protein concentration was measured by comparison with a known concentration of bovine serum albumin using a BCA Protein Assay Kit (Pierce Biotechnology, Rockford, IL, USA). A mixture of equal parts of an aliquot of protein and sample buffer with 10% 2-mercaptoethanol was subjected to 10% sodium dodecyl sulfate-polyacrylamide gel electrophoresis. The separated protein was then transferred onto a polyvinylidene difluoride membrane (Immobilon-P; Millipore). For immunoblotting, the following primary antibodies were used: Monoclonal Anti-D1 Dopamine Receptor antibody (1∶500 dilution; Sigma Aldrich), Anti-Dopamine D2 Receptor (1∶1000 dilution; Millipore), Phospho-p44/42 MAPK (Erk1/2) (Thr202/Tyr204) (197G2) Rabbit mAb, p44/42 MAPK (Erk1/2) Rabbit pAb, anti-GAPDH Rabbit mAb (1∶1000 dilution; Cell signaling, Danvers, MA, USA), and monoclonal anti-β-actin (1∶5000 dilution; Sigma Aldrich). The secondary antibody was as follows: HRP-conjugated goat anti-mouse IgG, HRP-conjugated goat anti-rabbit IgG (1∶2000 dilution; Pierce Biotechnology). Immunoreactive bands were visualized using Immuno Star® LD (Wako). The band intensity was measured using a LUMINESCENT IMAGE ANALYZER LAS-4000 UV mini (Fujifilm, Tokyo, Japan) and Multi Gauge Ver. 3.0 (Fujifilm). The protein levels of total or phosphorylated ERK1/2 were analyzed at the same time. For a quantitative analysis, total proteins were used as loading controls for phosphoprotein signals.

### Statistical analysis

Data are presented as mean ± standard error of the mean (S.E.M.). Statistical comparisons were made by Student's *t*-test, chi-square test, or one-way ANOVA followed by Dunnett's test using JSTAT software (Vector, Tokyo, Japan). Two-way ANOVA followed by post hoc one-way ANOVA with Bonferroni's multiple comparison test was used in analyzing data. Probability (p) values of less than 5% were considered statistically significant.

## Results

### DGKβ KO mice exhibited attention-deficit behaviors

**Figure 1:**
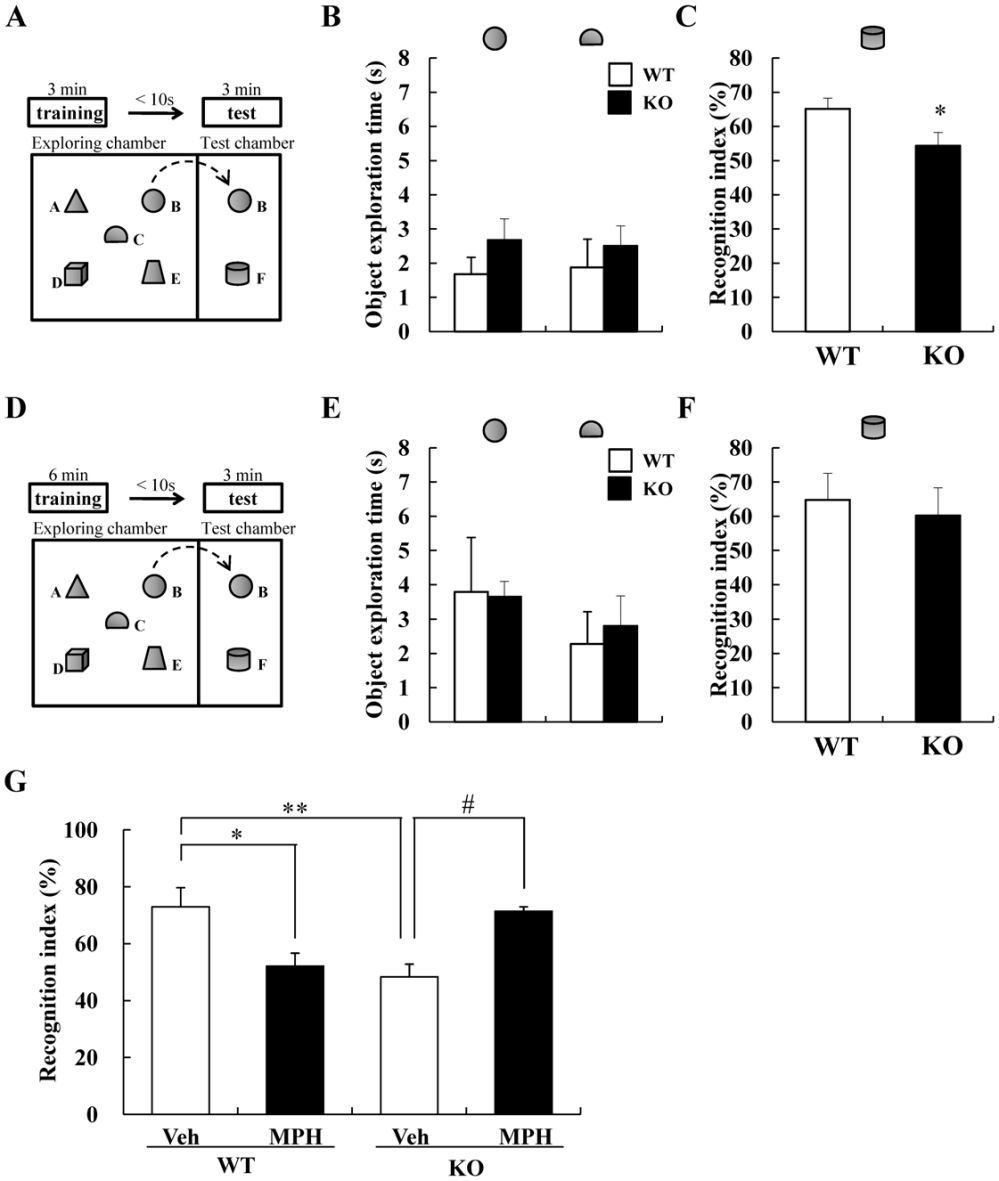
DGKβ KO mice showed an attention-deficit behavior in object-based attention test. (A) Mice were exposed to five objects for 3 min (training session), then, after an interval of 10 s, they were exposed to two objects that include a familiar and a novel objects for 3 min (retention session). (B) Object exploration time during the 3-min training session. (C) The novel-object discriminating abilities of mice were expressed as a recognition index. Values are expressed as the mean ± S.E.M. (KO: n = 8, WT: n = 9) *; p<0.01 vs. WT mice (*t*-test). (D) Mice were exposed to five objects for 6 min (training session), then, after an interval of 10 sec, they were exposed to two objects that include a familiar and a novel objects for 3 min (retention session). (E) Object exploration time during the 6-min training session. (F) The novel-object discriminating abilities of mice were expressed as a recognition index. Values are expressed as the mean ± S.E.M. (KO: n = 6, WT: n = 7). (G) The effect of MPH on the recognition index in retention phase after 3-min training phase. Values are expressed as the mean ± S.E.M. (n = 6, 7) *; p<0.05, **; p<0.01 vs. vehicle-treated WT mice, #; p<0.05 vs. vehicle-treated KO mice (Tukey's test).

To evaluate the attention-deficit behavior of DGKβ KO mice, we performed the object-based attention test, in accordance with previous report [21]. Firstly, mice were exposed to the five objects for 3 min (training session) and then, after an interval of 10 s, they were exposed to two objects that include a familiar and a novel objects for 3 min (retention session) (Figure 1A). There were no changes in the exploration time on each object during the training session (Figure 1B). In retention session, WT mice spent more time on the novel object, while DGKβ KO mice spent less time on the novel object (Figure 1C). But, when mice were exposed to the five objects for 6 min in training session (Figure 1D), in which WT and DGKβ KO mice spent equal time on each objects (Figure 1E), there was no difference in the time spent in the novel object during retention session (Figure 1F). In all retention sessions, WT and DGKβ KO mice spent equal time on exploring two objects (Figure S1). These results suggest that DGKβ KO mice showed attention-deficit behavior.

### The effect of methylphenidate (MPH) on the attention of DGKβ KO mice

Deficit of attention is a distinctive symptom of ADHD and ameliorated somewhat by therapeutic medicines such as MPH. To investigate whether attention-deficit behavior of DGKβ KO mice were ameliorated by MPH, we performed object-based attention test after MPH treatment. Throughout the experiment, MPH treatment decreased the time spent on exploring each objects (Figure S1E and F). In the WT mice, MPH treatment significantly decreased the recognition index, suggesting the attention-deficit behavior. On the other hand, MPH treatment significantly increased the recognition index of DGKβ KO mice (Figure 1G).

These results suggest that MPH improved the inattention of DGKβ KO mice.

### DGKβ KO mice were hyporesponsive to methylphenidate (MPH)-induced behavioral change

**Figure 2:**
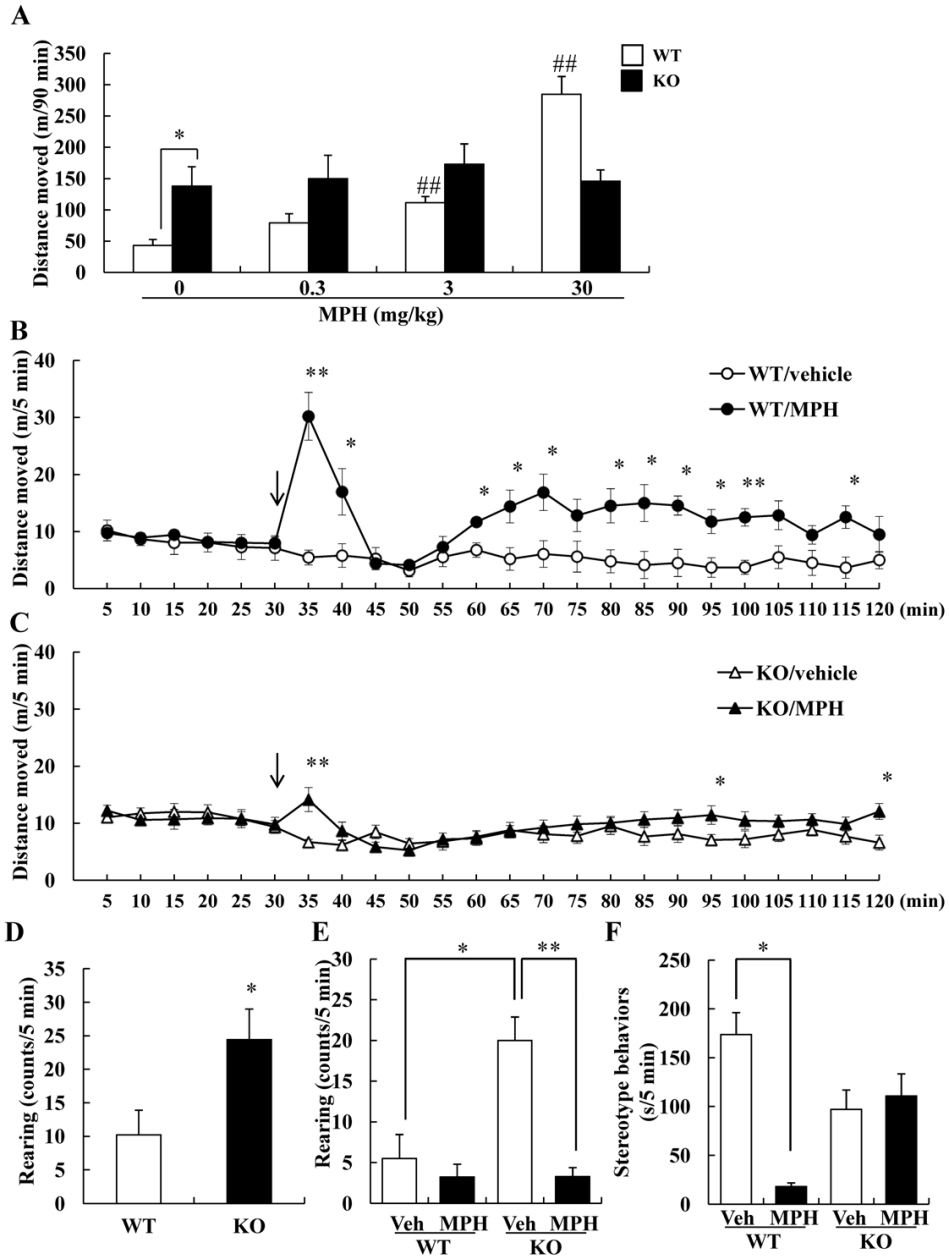
DGKβ KO mice showed an abnormal response to methylphenidate. (A) The locomotor activity after various doses of MPH. Each mouse was placed in a locomotor activity monitor for an initial period of 30 min and then injected with vehicle or MPH (0.3, 3, 30 mg/kg, i.p.). Horizontal activities of 90 min after drug treatment were recorded. Values are expressed as the mean ± S.E.M. (n = 5) *; p<0.05 vs. vehicle-treated WT mice (*t*-test), #; p<0.05, ##; p<0.01 vs. vehicle-treated WT mice (Dunnett's test). Each mouse was placed in a locomotor activity monitor for an initial period of 30 min (shown as arrow) and then injected with vehicle or MPH (30 mg/kg). Horizontal activity was recorded every 5 min for a 2-h period. Locomotor activity throughout the 2-h period of WT (B) and DGKβ KO (C) mice. Values are expressed as the mean ± S.E.M. (n = 4 to 10) *; p<0.05, **; p<0.01 vs. vehicle-treated group (*t*-test). (D) Rearing behavior of WT and DGKβ KO mice in the first 5 min of open field test. Values are expressed as the mean± S.E.M. (n = 5, 7) *; p<0.05 vs. WT mice (*t*-test). (E) Rearing behavior of MPH/vehicle treated WT and KO mice for 5 min after drug treatment. Values are expressed as the mean ± S.E.M. (n = 4 to 10) *; p<0.05, **; p<0.01 vs. vehicle-treated KO mice group (*t*-test). (F) The duration of the stereotyped behaviors for 5 min after drug treatment. Values are expressed as the mean ± S.E.M. (n = 4 to 10) *; p<0.05, **; p<0.01 vs. vehicle-treated WT mice group (*t*-test).

It is well known that MPH increase extracellular levels of dopamine, leading to an enhancement of locomotor activity in normal animals [22]. We administered various doses of MPH and investigated the change of locomotor activity for 90 min. Compared vehicle-treated WT mice with DGKβ KO mice, the locomotor activity of DGKβ KO mice was significantly increased. MPH increased the locomotor activity in WT mice, and it was significant at 3 and 30 mg/kg (i.p., MPH 0.3 mg/kg; p = 0.318, MPH 3 mg/kg; p = 0.005, and MPH 30 mg/kg; p = 0.003 vs. vehicle-treated WT mice). On the other hands, MPH did not increase the locomotor activity in DGKβ KO mice (MPH 0.3 mg/kg; p = 1.000, MPH 3 mg/kg; p = 0.958, and MPH 30 mg/kg; p = 1.000 vs. vehicle-treated KO mice) (Figure 2A). We also assessed the changes over time after MPH (30 mg/kg, i.p.) treatment. Administration of MPH to WT mice resulted in a marked increase in locomotor activity, followed by transient decrease (Figure 2B). Compared to the WT mice, in the DGKβ KO mice, MPH slightly increased the locomotor activity soon after MPH treatment (at the point of 35 min) (Figure 2C). We found significant effects of genotype [F(1, 24) = 9.01, p<0.01], treatment [F(1, 24) = 31.69, p<0.001], and genotype×treatment interaction [F(1, 24) = 14.32, p<0.001].

In this study, we measured rearing behavior, another stereotyped behavior in the novel environments. The number of rearing behaviors was significantly increased in DGKβ KO mice compared to WT mice in the novel environment (Figure 2D). The rearing behavior of vehicle-treated DGKβ KO mice remained higher than that of vehicle-treated WT mice 30 min after beginning experiment. MPH treatment significantly reduced the rearing behavior of DGKβ KO mice to an equal number of vehicle-treated WT mice (Figure 2E). We also investigated the duration of rearing behavior. They indicated a similar tendency, but no significance (p = 0.13) [WT (control): 11.47±5.16 s WT (MPH): 3.42±1.55 s KO (control): 38.54±9.88 s KO (MPH): 3.96±1.46 s]. Furthermore, we investigated the duration of stereotyped behavior other than rearing in the same time. The stereotyped behavior other than rearing of vehicle-treated DGKβ KO mice was less than that of vehicle-treated WT mice, but the difference was not significant (p = 0.056). After MPH treatment, the stereotyped behavior of WT mice was significantly reduced, but there were no changes in the DGKβ KO mice (Figure 2F). These results suggest that DGKβ KO mice showed low responsiveness to MPH.

**Figure 3:**
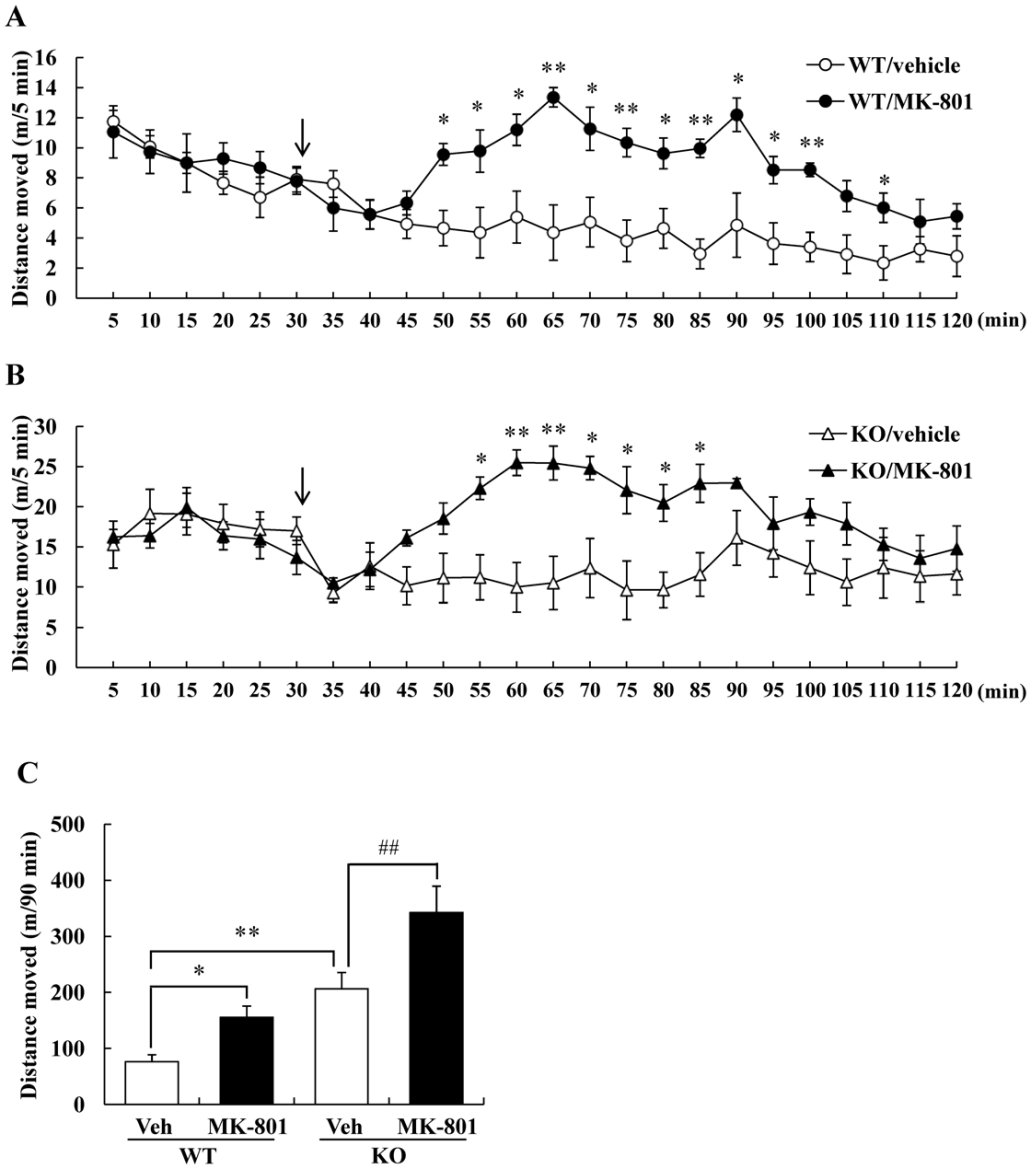
DGKβ KO mice showed normal responses to MK-801-induced hyperactivity. Each mouse was placed in a locomotor activity monitor for an initial period of 30 min (shown as arrow) and then injected with vehicle or MK-801 (0.3 mg/kg). Horizontal activity was recorded every 5 min for a 2-h period. Locomotor activity throughout the 2-h period of WT (A) and DGKβ KO (B) mice. Values are expressed as the mean ± S.E.M. (n = 4 or 5) *; p<0.05, **; p<0.01. (C) Total horizontal activities of 90 min after drug treatment were recorded. Values are expressed as the mean ± S.E.M. (n = 4 or 5) *; p<0.05, **; p<0.01 vs. vehicle-treated WT mice (*t*-test), ##; p<0.01 vs. vehicle-treated KO mice (*t*-test).

Not only the elevation of dopaminergic transmission, but also pharmacological disruption of the glutamatergic transmission was reported to modulate the locomotion of animals [23]–[24]. So, we assessed the psychostimulant effect of an *N*-methyl-d-aspartate (NMDA) receptor antagonist, MK-801 (dizocilpine) with the same method. In contrast to the case of MPH, locomotor activity in response to MK-801 treatment increased equally in both WT and DGKβ KO mice (Figure 3A–C).

### No changes occurred in dopaminergic neurons and receptors

**Figure 4:**
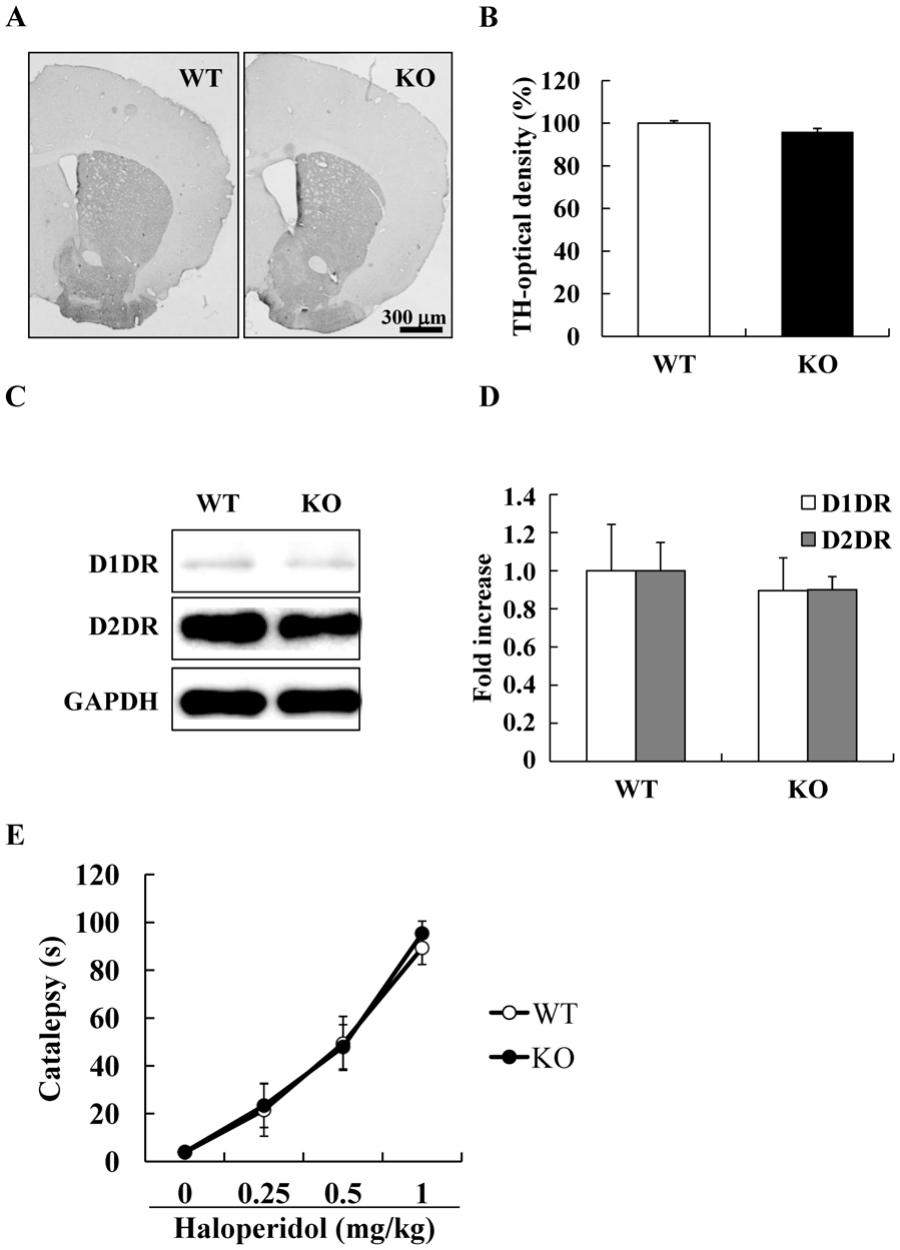
Dopaminergic systems in the striatum of the DGKβ KO mice. (A) Representative photograph of the coronal sections of the striatum immunostained for tyrosine hydroxylase (TH) of WT and DGKβ KO mice. Scale bar shows 300 µm. (B) Optical density of TH-positive fibers in the striatum of WT and DGKβ KO mice. Values are expressed as the mean ± S.E.M. (n = 3). (C) Representative immunoblots showing the expression levels of D1 dopamine receptor (D1DR) and D2 dopamine receptor (D2DR) in the striatum of WT and DGKβ KO mice. (D) Protein levels of D1DR and D2DR are quantified relative to the GAPDH levels. Values are expressed as the mean ± S.E.M. (n = 6). (E) The retention time of haloperidol-induced catalepsy of WT and DGKβ KO mice. Values are expressed as the mean ± S.E.M. (n = 6 to 12).

As DGKβ KO mice showed abnormal behavioral change against MPH, we hypothesized that changes in the dopamine system might have occurred in the DGKβ KO mice. In the striatum, no difference was noted in the density of TH-immunopositive fibers between WT and DGKβ KO mice (Figure 4A and B).

Previously, dysfunction of the dopamine D2 receptor has been reported in some genetically-modified animal models that showed lower responsiveness to methamphetamine-induced sensitization [25]. For this reason, we investigated the function of the dopamine D2 receptor, by measuring the degree of catalepsy induced by the dopamine D2 antagonist, haloperidol. Again, no difference was noted in the persistence time of catalepsy induced by haloperidol between WT and DGKβ KO mice (Figure 4E), suggesting that DGKβ KO mice had normal dopamine D2 receptor function. Furthermore, no changes were found in the relative protein expression of the dopamine D1 receptor or D2 receptor in the striatum (Figure 4C and D).

### The changes of phosphorylation levels of ERK1/2 after methylphenidate treatment

**Figure 5:**
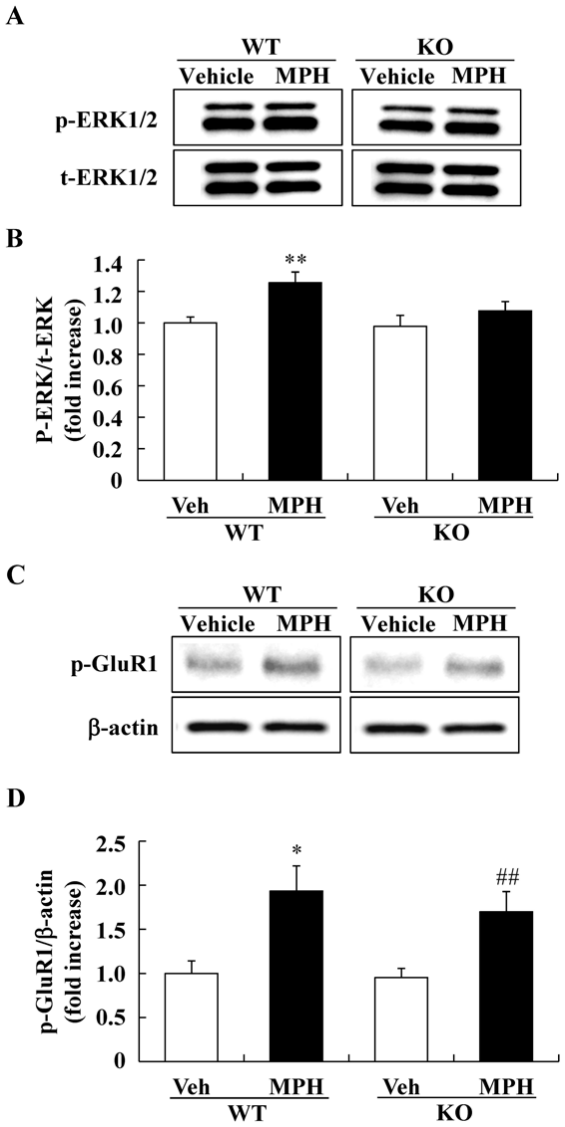
Western blot analysis of the phosphorylation levels of ERK1/2 and GluR1 in the striatum. Phosphorylated and total ERK1/2 levels in the striatum were measured by Western blot analysis. (A) Representative immunoblots showing the expression levels of phosphorylated ERK1/2 (p-ERK1/2) and total ERK1/2 (t-ERK1/2) in the striatum of WT and DGKβ KO mice 5 min after drug treatment. (B) Phosphorylation levels of ERK1/2 are quantified relative to the t-ERK1/2 levels. Values are expressed as the mean ± S.E.M. (n = 5 to 8) **; p<0.01 v.s. vehicle-treated WT mice group (*t*-test). (C) Representative immunoblots showing the expression levels of phosphorylated GluR1 (p-GluR1) in the striatum of WT and DGKβ KO mice 40 min after drug treatment. (D) Phosphorylation levels of GluR1 are quantified relative to the β-actin levels. Values are expressed as the mean ± S.E.M. (n = 6 to 8) *; p<0.01 vs. vehicle-treated WT mice group, ##; p<0.01 vs. vehicle-treated KO mice group (*t*-test).

Mouse locomotor behavior after psychostimulant treatment is known to be correlated with the phosphorylation levels of ERK1/2. For this reason, we also evaluated the phosphorylation level of ERK1/2 in the striatum of DGKβ KO mice. In the basal condition, no changes were noted in the phosphorylation levels of ERK1/2 in the striatum of DGKβ KO mice (WT; 1.00±0.04, KO; 1.02±0.12, n = 6). In contrast, 5 min after MPH treatment, the phosphorylation levels of ERK1/2 increased in the striatum of the WT mice, but not in DGKβ KO mice (Figure 5A and B). The levels of total ERK proteins were not changed by the MPH treatment in either genotype.

Furthermore, we investigated the phosphorylation levels of ERK1/2 in the hippocampus and prefrontal cortex at 5 min after MPH treatment to investigate whether MPH also affects the phosphorylation levels of ERK1/2 in these regions. In these regions, MPH did not affect the phosphorylation levels of ERK1/2 both in WT and KO mice (Figure S2). Previous report also showed that MPH had no effect on the phosphorylation levels of ERK1/2 in the prefrontal cortex [26]. These results suggest that the effect of MPH might be mainly mediated in the striatum.

We further investigated the cAMP-dependent pathway, which also plays a role in dopamine signaling. Five min after MPH treatment, there were no changes in the phosphorylation levels of GluR1 (Ser845) in the striatum of WT mice (data not shown). Forty min after MPH treatment, the phosphorylation levels of GluR1 (Ser845) was significantly increased in the striatum of WT mice. In the striatum of DGKβ KO mice, there were also increases of the phosphorylation levels of GluR1 (Ser845) (Figure 5C and D).

## Discussion

The purpose of the present study was to evaluate DGKβ KO mice as an animal model of ADHD. ADHD is characterized by inattention, impulsivity, and hyperactivity. In this study, it was revealed that DGKβ KO mice showed attention-deficit like behavior and hyperactive behavior which was especially pronounced in the familiar environment than in the novel environment. With regard to hyperactivity, phenotype of DGKβ KO mice appears similar to that of ADHD patients and spontaneously hypertensive rats [27]. In attention-deficit behavior of DGKβ KO mice, it was ameliorated by the MPH, which is commonly used as a treatment of ADHD. The effects of MPH against the locomotor activity of DGKβ KO mice were not similar to those of ADHD patients and general animal models of ADHD, other therapeutic agents for ADHD might show improving effects in DGKβ KO mice. These findings might suggest some involvements of DGKβ in the pathophysiology of ADHD. Unfortunately, to our knowledge, there is no report suggesting the mutations or polymorphisms in the DGKβ gene in ADHD patients. Further studies will be needed to make clear the involvement of DGKβ in ADHD.

To date, several behavioral tests were established to evaluate the animal attention. For example, in a 5-choice serial reaction time task, detections of attentional deficits of animal require training the subjects for long-term to enable them to acquire the behavioral skills [28]. Object-based attention test detects the deficit of attention of animals in a short time and usefulness of this test is recognized in p-chlorophenylalanine- or phencyclidine-treated mice [21]. Previously, we reported that hippocampally-based memory is affected in DGKβ KO mice [14]. Working memory and attention have much in common and are much dependent [21]. Therefore, there may be a possibility that hippocampal input is partly involved in this result.

MPH is a psychostimulant drug that exerts its pharmacological properties by blocking dopamine transporters and raising the extracellular concentration of dopamine in various brain regions [29]. MPH treatment was reported to cause significant decreases in the hyperactive locomotor activity of mice with dopamine transporter KO [18], casein kinase Iδ overexpression [19], or p35 KO [20]. In the present study, we used 30 mg/kg as the highest dose, which induced transient decreasing of locomotor activity after rapid increment in WT mice. This change over time of locomotor activity was seen in previous report [18]. MPH administration did not decrease locomotor activity of DGKβ KO mice, but the increment of locomotor activity was smaller than that seen in WT mice. Considering that DGKβ KO mice showed a normal response to MK-801, the absence of DGKβ did not affect the psychomotor stimulant response to the non-competitive NMDA receptor antagonist.

We also investigated the rearing behavior and other stereotyped behavior. Stereotyped behavior, such as rearing behavior, has been observed in a mouse model of psychiatric disorders [30]. In this study, DGKβ KO mice frequently showed rearing behavior. In some animal models of ADHD, the increment of vertical activities was also reported [19], therefore, increment of rearing behavior of DGKβ KO mice might be caused by its hyperactivity. The effect of psychostimulants on stereotyped behaviors of animals was reported by some researchers [31], [32]. In this study, we investigated stereotyped behaviors for a short time (5 min), and MPH treatment decreased them in WT mice, but not in DGKβ KO mice. It might be due to the dramatically increment of locomotor activity of WT mice in this time point.

Previously, three major striatal signaling pathways (protein kinase A/DARPP-32 [33]–[34], Akt/glycogen synthase kinase 3 [35], and ERK [36]–[37]) were reported to regulate dopamine-mediated locomotor activity. Of these, the ERK signaling in particular appears to play an important, but as yet undetermined, role in psychostimulant stimulus responses [18]. Under basal conditions, no differences in the expression level or functions of dopamine receptors were observed between WT and DGKβ KO mice. In addition, no changes were noted in the levels of ERK1/2 phosphorylation in the striatum when WT and DGKβ KO mice were compared. These results suggest that the hyperactive phenotype of DGKβ KO mice is not due to the dopaminergic signal in the striatum. In the present study, we found no changes in phosphorylation levels of ERK in the striatum of DGKβ KO mice following MPH treatment.

It has been reported that methylphenidate affects dopamine/DARPP-32 signaling in adult [38]. Previous work has shown that, in dopamine D1 receptor KO mice and Thr-34→Ala DARPP-32 (T34A) mutant mice, no ERK activation occurred in the striatum following *d*-amphetamine treatment [39], suggesting that dopamine D1 receptor/DARPP-32 signal activate ERK following a stimulus by a phychostimulant drug. DGKs, which convert DG to PA, are located downstream of Gq-protein [5]. Dopamine D2 agonists are thought to activate ERK in striatal slices via coupling of a Gq-protein to phospholipase C β pathway and mobilization of intracellular calcium stores [40].

Although dopamine D1 and D2 receptors are classically considered to be localized in distinct striatal subpopulations [41], some reports suggests a synergism of both D1 and D2 receptors in the same striatal subpopulation [37]. This would mean that both dopamine D1 and D2 receptors are involved in ERK phosphorylation and that DGKβ regulates the ERK phosphorylation. However, MPH also affect the norepinephrine transporters [42], so, further study would need to examine the upstream factors of ERK after MPH treatment.

Previously, it was reported that some DGK isozymes (α, ζ, and ι) influence the signaling of Ras, an upstream molecule of the B-Raf/C-Raf/MEK (mitogen-activated protein kinase/extracellular signal-regulated kinase kinas)/ERK pathway, by modulating activities of Ras guanyl nucleotide-releasing protein [43]–[45]. Interestingly, it was revealed that DGKη regulates B-Raf/C-Raf/MEK/ERK signaling cascade acting as scaffold/adaptor protein [46]. Although the structure, expression pattern, and function of DGKs were diverse and each has its own biological functions, there is a possibility that DGKβ also involved in the regulation of B-Raf/C-Raf/MEK/ERK signaling cascade. However, further studies are needed to clarify the detailed mechanism that underlies DGKβ regulation of ERK phosphorylation.

In conclusion, DGKβ KO mice showed attention-deficit behavior and abnormal response to MPH-induced hyperactivity. These results suggest the involvement of DGKβin the pathophysiology of ADHD. Because DGKβ is downstream signals of various receptors, there is a possibility that various signaling is affected at the same time. Further studies on the functions of DGKβ may lead to clarification of the mechanisms of many neurological diseases.

## Supporting Information

Figure S1 **The time spent exploring each objects in the retention test of object-based attention test.** (A) Object exploration time during the retention test after 3-min training session. (B) Total exploration time during the retention test after 3-min training session. Values are expressed as the mean ± S.E.M. (KO: n = 8, WT: n = 9). (C) Object exploration time during the retention test after 6-min training session. (D) Total exploration time during the retention test after 6-min training session. Values are expressed as the mean ± S.E.M. (KO: n = 6, WT: n = 7). (E) Object exploration time of vehicle or MPH treated mice during the retention test after 3-min training session. (F) Total exploration time of vehicle or MPH treated mice during the retention test after 3-min training session. Values are expressed as the mean ± S.E.M. (n = 6, 7) *; p<0.05 vs. vehicle-treated WT mice, #; p<0.05 vs. vehicle-treated KO mice.
(TIFF)

Figure S2 **Western blot analysis of the phosphorylation levels of ERK1/2 in the prefrontal cortex and hippocampus.** Phosphorylated and total ERK1/2 levels in the prefrontal cortex were measured by Western blot analysis. Representative immunoblots showing the expression levels of phosphorylated ERK1/2 (p-ERK1/2) and total ERK1/2 (t-ERK1/2) in the prefrontal cortex of WT and DGKβ KO mice 5 min after (A) and 40 min (B) after drug treatment. Representative immunoblots showing the expression levels of phosphorylated ERK1/2 (p-ERK1/2) and total ERK1/2 (t-ERK1/2) in the hippocampus of WT and DGKβ KO mice 5 min after (C) and 40 min (D) after drug treatment. Values are expressed as the mean ± S.E.M. (n = 6).
(TIFF)
