# Supplementary figures and images for: Diacylglycerol Kinase β Knockout Mice Exhibit Attention-Deficit Behavior and an Abnormal Response on Methylphenidate-Induced Hyperactivity
Source: PLoS One. 2012 May 10;7(5):e37058. doi: 10.1371/journal.pone.0037058 (PMC3349656; doi:10.1371/journal.pone.0037058)

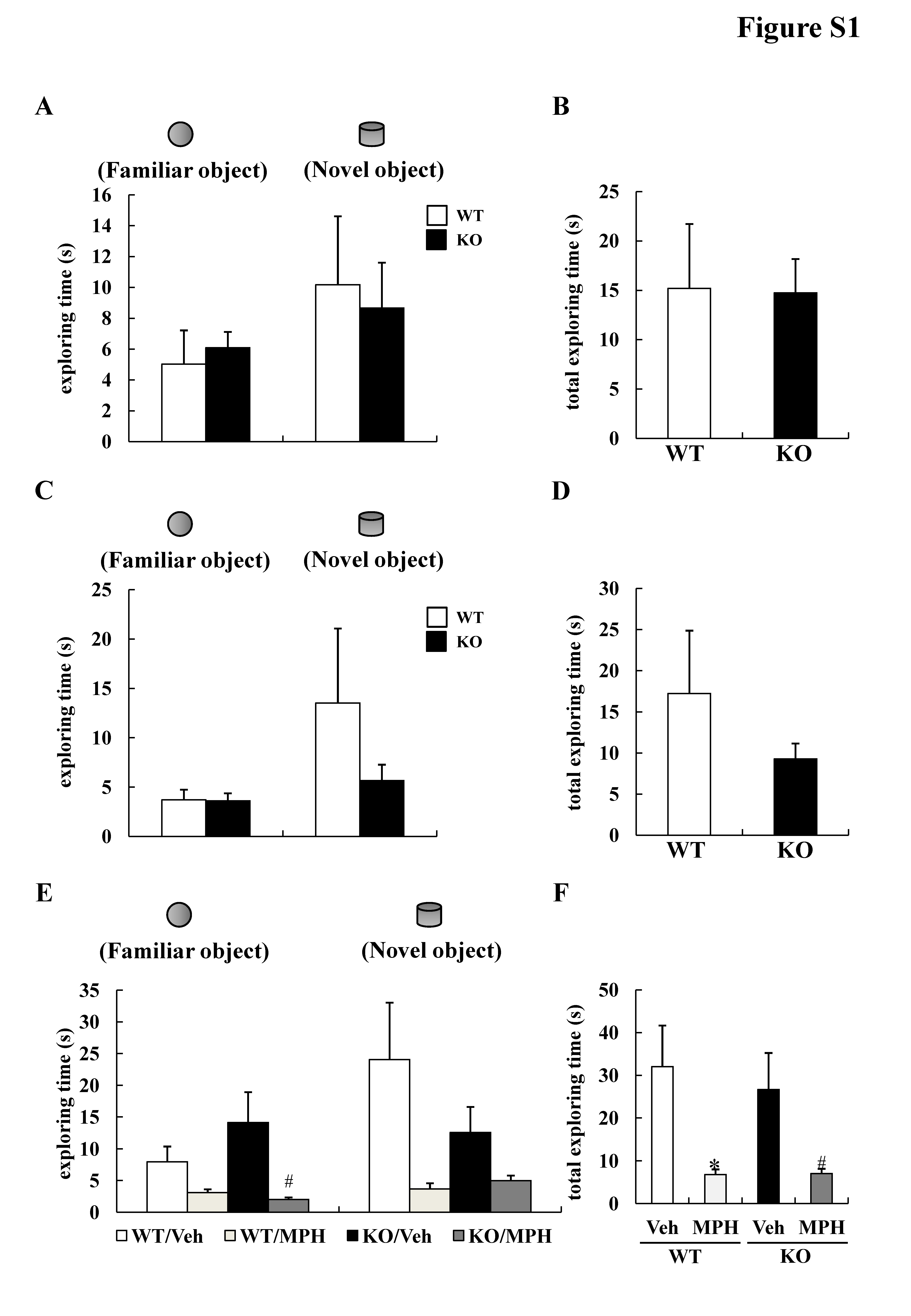

Supplement: Figure S1 — The time spent exploring each objects in the retention test of object-based attention test. (A) Object exploration time during the retention test after 3-min training session. (B) Total exploration time during the retention test after 3-min training session. Values are expressed as the mean ± S.E.M. (KO: n = 8, WT: n = 9). (C) Object exploration time during the retention test after 6-min training session. (D) Total exploration time during the retention test after 6-min training session. Values are expressed as the mean ± S.E.M. (KO: n = 6, WT: n = 7). (E) Object exploration time of vehicle or MPH treated mice during the retention test after 3-min training session. (F) Total exploration time of vehicle or MPH treated mice during the retention test after 3-min training session. Values are expressed as the mean ± S.E.M. (n = 6, 7) *; p<0.05 vs. vehicle-treated WT mice, #; p<0.05 vs. vehicle-treated KO mice. (TIFF) [file pone.0037058.s001.tiff]

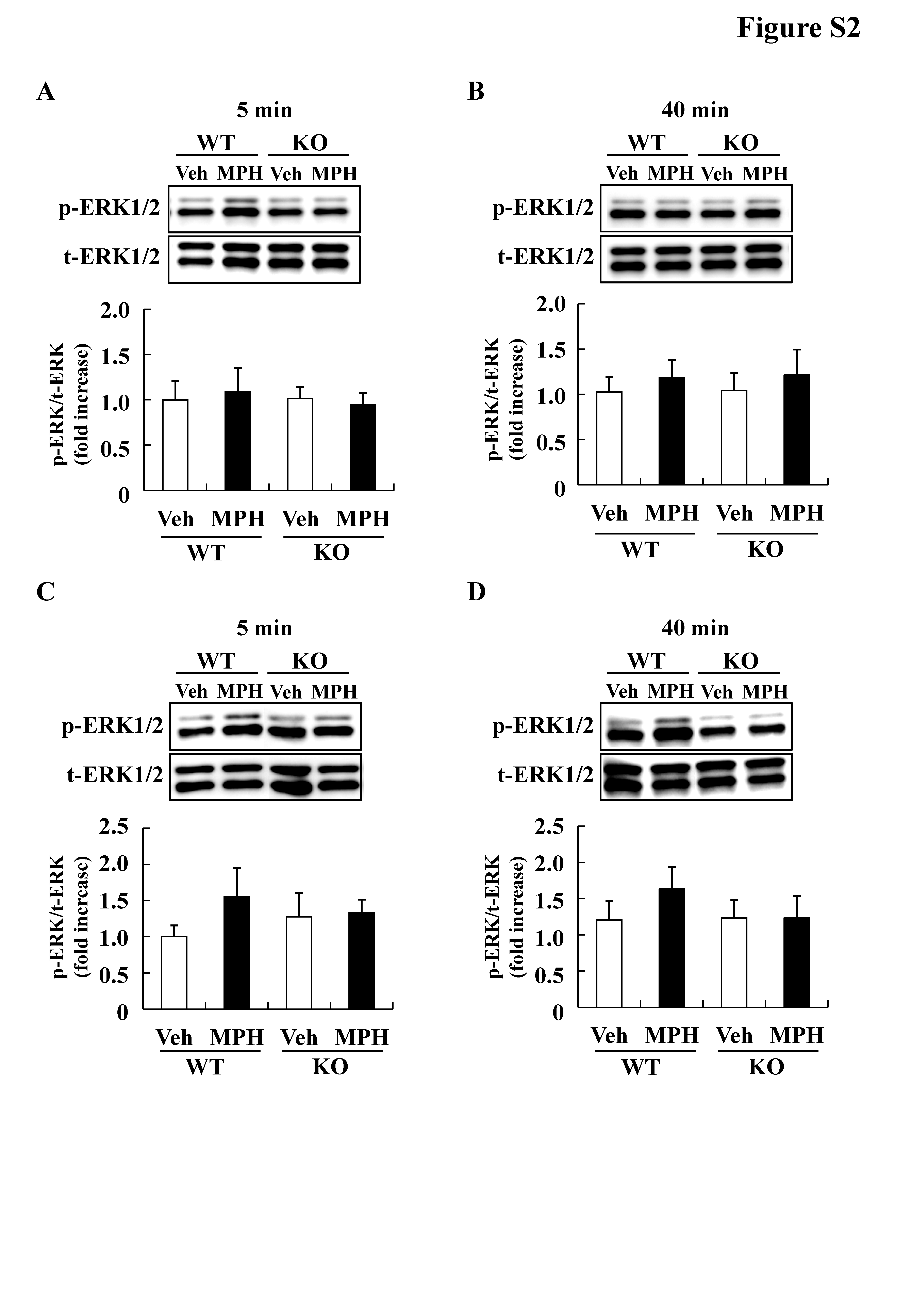

Supplement: Figure S2 — Western blot analysis of the phosphorylation levels of ERK1/2 in the prefrontal cortex and hippocampus. Phosphorylated and total ERK1/2 levels in the prefrontal cortex were measured by Western blot analysis. Representative immunoblots showing the expression levels of phosphorylated ERK1/2 (p-ERK1/2) and total ERK1/2 (t-ERK1/2) in the prefrontal cortex of WT and DGKβ KO mice 5 min after (A) and 40 min (B) after drug treatment. Representative immunoblots showing the expression levels of phosphorylated ERK1/2 (p-ERK1/2) and total ERK1/2 (t-ERK1/2) in the hippocampus of WT and DGKβ KO mice 5 min after (C) and 40 min (D) after drug treatment. Values are expressed as the mean ± S.E.M. (n = 6). (TIFF) [file pone.0037058.s002.tiff]
